# Assessment of fluralaner as a treatment in controlling *Dermanyssus gallinae* infestation on commercial layer farms and the potential for resulting benefits of improved bird welfare and productivity

**DOI:** 10.1186/s13071-021-04685-7

**Published:** 2021-03-31

**Authors:** Ivo Petersen, Katharina Johannhörster, Eric Pagot, Damian Escribano, Eva Zschiesche, Déborah Temple, Emmanuel Thomas

**Affiliations:** 1 MSD Animal Health Innovation GmbH, Zur Propstei, 55270 Schwabenheim, Germany; 2 Praxis Dr. Pöppel, Drubbelstrasse 2, 33119 Delbrück, Germany; 3 Centre Technique Des Productions Animales, Zoopole Dévelopment, 2 rue Jean Rostand, 22440 Ploufragan, France; 4 Interdisciplinary Laboratory of Clinical Analysis (Interlab-UMU), Veterinary School, Campus of Excellence Mare Nostrum, University of Murcia, Campus de Espinardo s/n, 30100 Murcia, Espinardo Spain; 5 Animal Production Department, Regional “Campus of Excellence Mare Nostrum”, University of Murcia, 30100 Murcia, Espinardo Spain; 6 School of Veterinary Medicine, Universitat Autonoma de Barcelona, Bellaterra, Spain

**Keywords:** *Dermanyssus gallinae*, Fluralaner, Hen welfare, Hen health, Isoxazoline, Poultry red mite, Layer hens

## Abstract

**Background:**

Poultry red mite (PRM) (*Dermanyssus gallinae*) infestations are a cause of anaemia, impaired productivity and stress-related behaviours linked to reduced hen welfare. A study investigated the potential health, welfare and productivity benefits following fluralaner treatment to eliminate PRM from infested hens.

**Methods:**

A PRM-infested layer house was selected on a free-range farm (5400 hens) and an aviary farm (42,400 hens). Fluralaner (Exzolt^®^; 0.5 mg/kg body weight) was administered twice, 7 days apart (Weeks 0 and 1), via drinking water. Mite populations were monitored by traps. Cameras recorded nighttime hen behaviours weekly, pre- and post-treatment. On the free-range farm, daytime behaviours were also recorded weekly. For pre- and post-treatment corticosterone assessments, eggs were randomly collected on both farms, and blood samples were collected from 50 randomly selected aviary farm hens. Production parameters were assessed using farm records.

**Results:**

Throughout the post-treatment period, fluralaner efficacy against PRM was > 99% on both farms. On the aviary and free-range farms, treatment was followed by significant nighttime increases in the proportion of resting hens (*P* < 0.0001; *P* = 0.0175, respectively). Significant post-treatment versus pre-treatment nighttime reductions were observed in head shaking (aviary, *P* < 0.0001; free-range *P* = 0.0233) and preening (*P* = 0.0032; *P* = 0.0018) and on the aviary farm in bouts of body shaking (*P* = 0.0108), vertical wing shaking (*P* = 0.0002), head scratching (*P* = 0.0335), and gentle feather pecking (*P* < 0.0001). On the free-range farm there were significant daytime reductions in head scratching (*P* < 0.0001), head shaking (*P* = 0.0492) and preening (*P* = 0.0012). Relative to standard production parameters, no differences were detected on the aviary farm, but on the free-range farm the laying rate decline with increasing age was less than expected and the increase in egg weight greater than expected. Post-treatment increases in egg and plasma corticosterone were suggestive of stress factors in addition to mite infestation. Red blood cell counts and haematocrit increased following treatment.

**Conclusion:**

Fluralaner treatment eliminated mite challenge, leading to improved hen welfare and health, based on reductions in stress-related behaviours and restoration of the anaemia-inducing effects of mite blood feeding. 
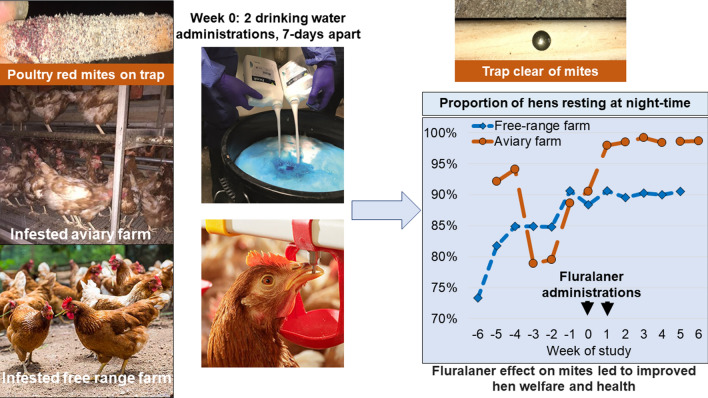

## Background

Infestations with *Dermanyssus gallinae*, the poultry red mite (PRM), an obligatory blood feeder, have a negative effect on all types of poultry production, ranging from backyard and organic farms to intensive, enriched cage or barn systems [1]. Anaemia can be a consequence of heavy infestations, and the PRM has been shown to be a vector of important viral and bacterial pathogens, including *Salmonella enteritidis* and avian influenza virus [2–4]. Laboratory infestations of poultry have been found to be a cause of stress and impaired welfare, manifested by behavioural changes such as an increase in grooming and head scratching [5], increased levels of plasma corticosterone, adrenaline and β-globulins, and reductions in γ-globulins in infested hens compared to uninfested controls [6, 7]. Thus, the findings of moderate somatic and high psychogenic stress factors (corticosterone secretion and adrenaline secretion, respectively) in infested birds indicate that PRM infestations are a cause of impaired welfare, which in turn can lead to impaired productivity.

Annual PRM control and production losses in laying hens was estimated in a 2005 paper to be €130 million [8]. In 2021 terms this is likely to be an underestimate because: Infestation rates in European flocks are now believed to be much higher [4]; until the 2017 introduction of fluralaner there was a declining efficacy in treatments [3, 4]; for safety reasons many treatments that were used have been withdrawn [3, 4]; perhaps associated with those changes, a larger proportion of flocks are now believed to be infested (in 2013, in some European countries, flock infestation rates were reported at 100%) [8]. Additionally, there has been a substantial increase in the number of laying hens in Europe [4]. These losses are attributed to the negative impact of PRM infestations on the feed conversion ratio, egg production, proportion of downgraded eggs, susceptibility to intercurrent disease and mortality rate [1, 3, 4, 9]. The problem of flock infestations with the PRM has been exacerbated by the difficulties of implementing measures that are effective in the face of increasing resistance to registered chemicals, while being safe for birds, workers and the environment and avoiding egg and meat residues that could have a detrimental effect on human health [1, 4, 10, 11].

Fluralaner is an isoxazoline compound that is approved for the treatment of poultry red mite infestations by two drinking-water administrations with a 7-day interval and having egg and meat withholding times of 0 and 14 days, respectively [12]. In vitro studies have demonstrated that field isolates of the northern fowl mite (*Ornithonyssus sylviarum*) and the PRM that are highly sensitive to fluralaner are relatively insensitive to other compounds, including spinosad, phoxim, propoxur and deltamethrin [13, 14]. The mite-killing activity of fluralaner is evident within 4 h of the first administration and is maintained for at least the next 14 days [15]. Adult mites are killed quickly, while any egg or non-feeding larvae present at the time of treatment are subsequently killed as they mature to nymphs and take a blood meal, with mite populations reported to decline by as much as 99% within 3 days of the first fluralaner administration and up to 100% within 2 days of the second administration [16]. Elimination of the mites in this way may reduce or remove the welfare and productivity effects of infestations [4, 16, 17]. A study was conducted in Europe to generate further evidence of the potential benefits of elimination of PRM infestations.

## Methods

The study was conducted on two commercial egg production layer sites, one free-range farm in Germany housing LSL hens, the other an aviary farm in France with Lohmann Brown hens. The objective was to assess the effects of mite elimination by an acaricidal treatment with fluralaner on behavioural welfare parameters in chickens naturally infested with *D. gallinae*. The potential benefits of mite elimination on bird health and productivity were monitored by comparing pre- and post-treatment parameters and by assessing production parameters for each flock against the published standards for the age and breed of hen on each farm. All procedures were in alignment with the principles of Good Clinical Practice VICH GL9 (GCP) [18]. An informed consent was completed by the farm owners prior to any enrolment and initiation of treatment.

To qualify for the study, a farm had to have a known history and current presence of PRM infestation, along with a facility in which a PRM challenge could be monitored, to have suitable equipment for accurate delivery of fluralaner via drinking water (dosing pump or medication tank) and to accept the installation of cameras to monitor bird behaviours. On each farm, a single house was used for the study. To avoid cross-contamination to the study house, the farmer was required to accept fluralaner administrations of all other farm houses concurrently with study bird administrations, and to avoid use of any acaricidal product, other than the scheduled fluralaner treatment, during the 2 weeks prior to starting and throughout the study period.

The study was initiated with pre-treatment observations during July and August of 2018 in the free-range and aviary farms, respectively, and concluded in September and October of the same year. The study house on the free-range farm was naturally ventilated, with both natural and artificial light from 04:00 until 20:00 and contained 5400 chickens, with a mean body weight of 1.8 (± 0.1) kg. Birds were allowed to roam outside during daytime. On the aviary farm, the study house contained 42,400 birds, 66 weeks old with a mean body weight of 1.8 ± 0.2 kg at Week − 5. For both farms, feed was provided according to standard procedure and water was provided ad libitum. Formal health assessments of study birds were completed by a veterinarian at weekly intervals, and general observations were made by farm staff throughout the study, with any abnormal observations to be notified to the veterinary investigator.

### Treatment

At the time of initial treatment, in August on the free-range farm and September on the aviary farm, birds were 62 and 71 weeks of age, respectively. Before treatment it was ensured that the drinking water system worked and was free of leaks and that there was no other drinking water source available. The fluralaner solution (Exzolt^®^, MSD Animal Health, Germany) was administered according to label directions to achieve a dose rate of 0.5 mg/kg body weight, twice with a 7-day interval (Weeks 0 and 1) (Table 1) [12]. The required volume of product was calculated using the body weights of 20 representative birds to estimate the total body weight of the group of chickens to be treated. That volume was added to 1 day’s water consumption, based on farm records of the previous day’s consumption. On the aviary farm a review of records indicated that an overestimation of the number of hens in the study house resulted in the labelled dose rate being exceeded by 11%. The duration of treatment administration ranged from 7 h 45 to 10 h.

**Table 1:** Schedule of study activities

Data collected	Units	Study week												
		− 6	− 5	− 4	− 3	− 2	− 1	0	1	2	3	4	5	6
Mite traps	≥ 20 traps	X^a^	X^b^	X^b^	X^b^	X^b^	X	X	X^b^	X	X^b^	X^b^	X^b^	X
Treatment^d^	House							X	X					
Observations														
Behaviour (day)	Video recording	X^a^	X^a^	X^a^		X^a^	X^a^	X^a^	X^a^	X^a^	X^a^	X^a^	X^a^	
Behaviour (night)	Video recording^c^	X^a^	X	X	X^b^	X	X	X	X	X	X	X	X	X^b^
Health	100 birds	X^a^	X	X	X	X	X	X	X	X	X	X	X	X
Samplings														
Blood	*n* = 50 (randomly selected in flock)				X^b^		X^b^						X^b^	
Eggs	*n* = 40 (randomly selected)	X^a^			X^b^		X^b^	X^a^					X^b^	X^a^
Production	House	X^a^	X	X	X	X	X	X	X	X	X	X	X	X

^a^Free-range farm only

^b^Aviary farm only

^c^Two cameras used on aviary farm

^d^First fluralaner administration, 2 or 3 days before trap placement, 3 or 4 days before behavioural observations (Weeks 0 and 1), 1 day before blood sampling (Week 1)

### Assessments of challenge with *Dermanyssus gallinae*

To determine the level of mite challenge, PRM traps (Avivet) were used. These traps comprised a black Tylene tube, 50 mm in length, with inner and outer diameters of 12 and 16 mm, respectively. The tube contains rolled corrugated cardboard. These commercially available traps have been validated for quantitative assessment of PRM infestations and were placed in the study houses according to the manufacturer’s guidance [19]. On the free-range farm, 20 traps were placed near the lowest pole of the perches, above the manure pits, in Weeks − 6, − 1, 0 and 6. On the aviary farm, 24 traps were placed under perches at weekly intervals from Week -5 to Week 6. After 1 day (free-range farm) or 2 days (aviary farm), traps were collected and individually sealed in small plastic bags, each of which was then placed in a large plastic bag and stored at − 18 to − 20 °C or colder before being sent to a laboratory with experience in identifying mites by species and in counting and differentiating mite stages. The mites in each trap and its plastic bag were poured into a Petri dish, and mites or eggs remaining on the cardboard of the trap or in the plastic bag were thoroughly collected and added to the mites in the dish. Mite eggs, larvae and nymphs and adults were differentiated and counted separately. In traps with up to 250 mg of mites (total weight of eggs and mobile stages), all *D. gallinae* were differentiated and counted. If the weight of collected mites exceeded 250 mg, a subsample of approximately 100 mg was used.

### Behavioural observations

On each farm, two cameras were used to monitor hen behaviours once per week. On the free-range farm, one camera recorded daytime behaviours, the other (infra-red) nighttime behaviours. Two infra-red cameras were used on the aviary farm, where only nighttime behaviours were monitored. Selection of the observation field for each camera was based on the expected number of birds present during the observation times; approximately 50 birds were observed at the aviary farm and approximately 30 birds at the free-range farm. Each recording began 3 h after the onset of darkness, when mites are most active, at the same time on the same day in each week, beginning at Week − 6 (free-range farm) or − 5 five (aviary farm) and continuing to Weeks 5 and 6, respectively. All camera recordings were evaluated by a single trained observer. Hens were scored, higher scores indicating greater stress, for the behaviour categories of body shaking, vertical wing shaking, gentle feather pecking, severe feather pecking, aggression, head shaking, head scratching and preening [17]. Behavioural categories were mutually exclusive. Observations included continuous recordings of each targeted behaviour every 2 min for 1 min over a 60-min period and scan samplings of hen resting time. The percentage of resting hens at each observation point was expressed in proportion to the total number of observed hens. The number of events for a behaviour was expressed as the number of bouts (number of times a behavioural element was observed) per hen within 15 min in proportion to the total number of hens observed. The number of resting and active hens was assessed by scan sampling every 2 min. Each category was measured as the number of events or animals performing the behaviour.

### Haematology and stress indicators

Blood samples for complete blood counts and blood chemistry measurements were collected during Weeks − 2, 0 and 6 from 40 randomly selected hens on the aviary farm. On the aviary farm, 40 eggs were randomly collected during Weeks − 3, − 1 and 6 and on the free-range farm during Weeks − 6 and 0, and 31 eggs were collected on this farm during Week 6. Corticosterone concentrations in blood samples were estimated from hens on the aviary farm and in egg samples from both study farms. The red blood cells, haematocrit, haemoglobin concentration, mean corpuscular volume, mean corpuscular haemoglobin and mean corpuscular haemoglobin concentrations were measured using an automated hematology analyzer (ADVIA 120 hematology system, Siemens Healthineers, Spain). Stained blood smears (Diff-Quik) were examined under 100× light microscopy [20] to manually count 60 white blood cells and determine the heterophil to lymphocyte (H:L) ratio. Heparin tubes were centrifuged for 10 min at 3000 g to obtain plasma. Corticosterone levels of egg extracted albumin and yolk and also of the plasma samples were measured by a high-sensitivity EIA kit (Corticosterone HS [High Sensitivity] EIA, IDS^®^ Immunodiagnostic Systems, Boldon, UK) following the manufacturer's instructions. The extraction procedure for egg albumin and yolk corticosterone used a procedure previously described by Cook et al. [21], and results were expressed as nanograms per gram of the freeze-dried sample taken for extraction. All tests were performed at the Interdisciplinary Laboratory of Clinical Analysis (Interlab-UMU, University of Murcia, Spain).

### Production parameters

Farm production records were accessed to determine the hen weekly mortality rate (%), mean (%) laying rate and mean egg weights for each study week.

### Statistical analysis

Statistical units were: the trap for the assessment of PRM infestation, the house for general health observations and performance evaluation, the observational point for behavioural evaluation, the individual animal for assessment of body weight and blood sampling and health status evaluation, and the egg for egg collection and corticosterone determination.

The antiparasitic efficacy was calculated for each post-treatment time point using the formula:

$$ \% {\text{efficacy}}\; = \;\frac{{X_{{{\text{pre}}}} \; - \;X_{{{\text{post}}}} }}{{X_{{{\text{pre}}}} }}\;.100 $$

where *X*_pre_ is the arithmetic mean pre-treatment mite (mobile stages: larvae, nymphs, adults) count per trap, and *X*_post_ is the mean post-treatment mite count per trap. Product efficacy was claimed if the percentage efficacy exceeded 90% and if the post-treatment mite counts were significantly less than pre-treatment counts (two-sample *t*-test).

Changes in production data (weekly mortality rate, mean laying rate and mean egg weight) were analysed for significant changes over time using a linear regression model. The slope of the regression line was compared to zero, a positive slope indicating an increase with time, a negative slope a decrease with time. Laying rates and egg weights were assessed in relation to published standard production data for the breed of bird used on each farm [22, 23]. Pre- and post-treatment behavioural observations were compared using a mixed linear model. The number of bouts (or % activity of hens) was the dependent variable; study phase (pre- or post-treatment) and, for the free-range farm, day and night observation point interactions were the main effects to be investigated; study week was the repeated factor and observation point the random factor. The physiological parameters were analysed by comparing each post-treatment value with the pretreatment value using a two-sided two-sample *t*-test. The statistical tests were conducted using a threshold of *α* = 0.05.

## Results

There were no adverse events reported from either farm following treatment.

### Counts of *Dermanyssus gallinae*

**Fig. 1:**
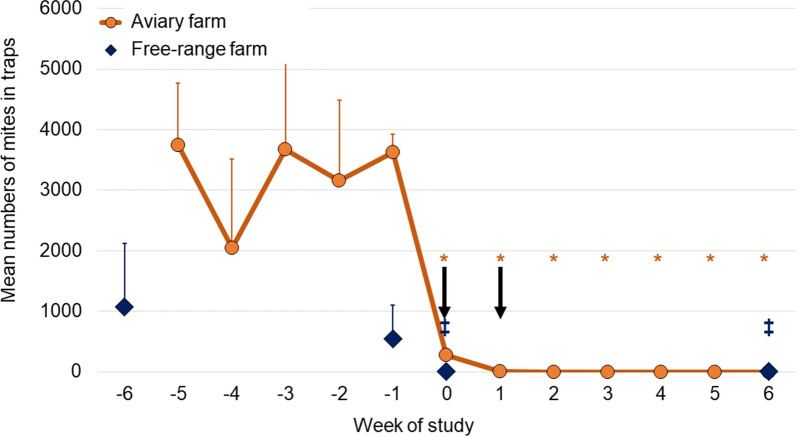
Arithmetic mean and standard deviation of *Dermanyssus gallinae* counts from traps placed weekly on the aviary farm and during Weeks − 6, − 1, 0 and 6 on the free-range farm (aviary farm, Weeks 0 through 6 versus Week − 1, *P < 0.0001); free-range farm (points with no line, Weeks 0 and 6 versus Week − 1, ^‡^*P* = 0.0014). Arrows indicate fluralaner administrations (Weeks 0 and 1)

Prior to treatment, the mite counts on the aviary farm comprised approximately 70% adults and 30% nymphs and on the free-range farm approximately equal proportions of adults and nymphs, with only a few larvae (≤ 1% of motile mite life stages) identified on each farm. On both farms, there was a sharp and statistically significant decrease in mite counts following the first administration of fluralaner (Fig. 1). Efficacy was 100% by Week 1 on the aviary farm (*P* < 0.0001) and remained at that level until Week 6 when a single mite was identified in one trap, while on the free-range farm the reductions in mite counts following treatment were > 99% at Week 0 (*P* = 0.0014) and at Week 6 (*P* = 0.0014), when the mean mite count was 1.4 (range 0–19). The antiparasitic efficacy of fluralaner against blood-feeding stages of *D. gallinae* was therefore achieved, and the post-treatment challenge to the birds is considered to have been eliminated or reduced to clinically irrelevant levels.

### Behavioural observations

**Fig. 2:**
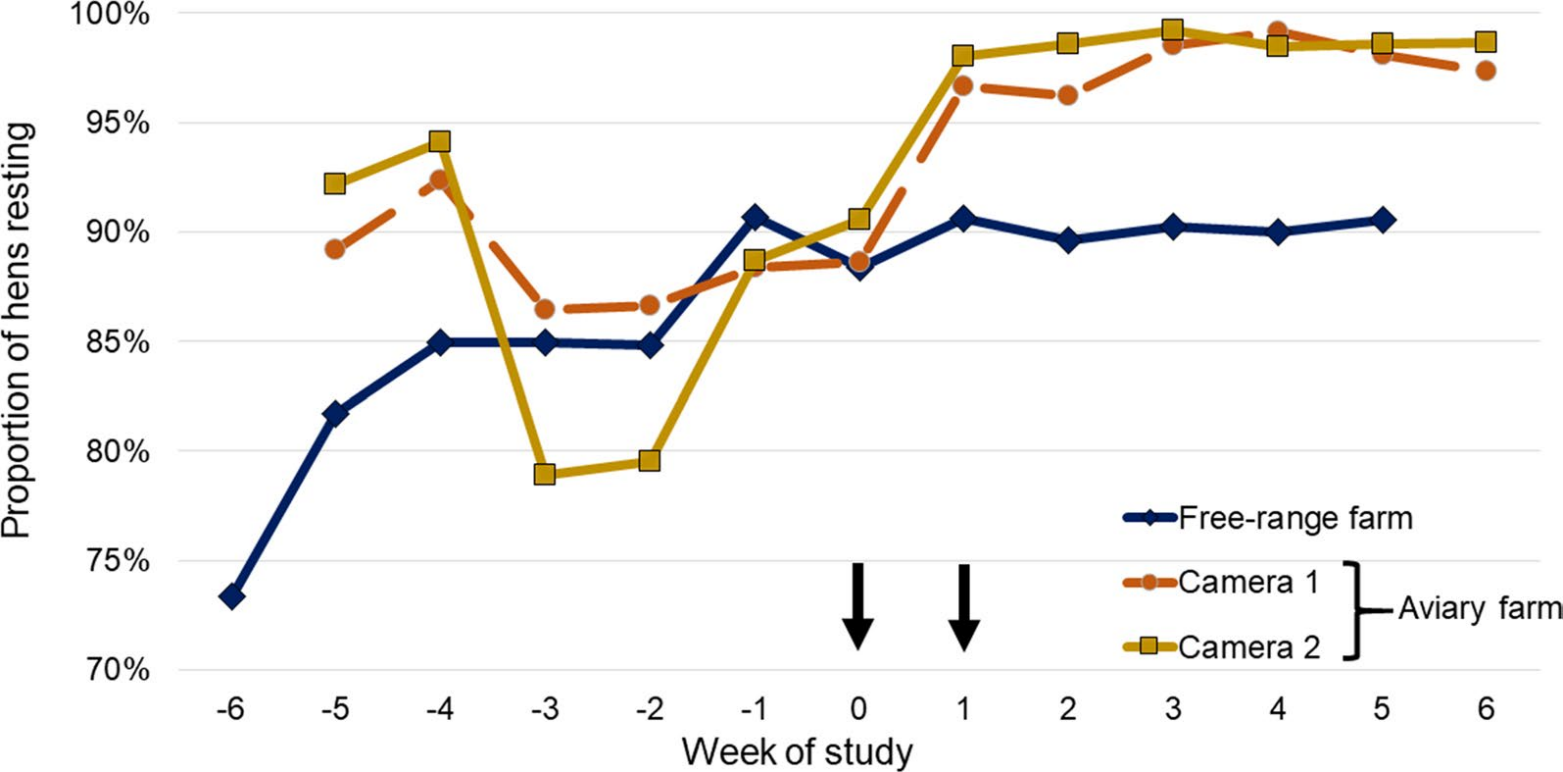
Percentage of resting hens observed in nighttime video recordings for each observation point. Arrows indicate fluralaner administrations (Weeks 0 and 1)

**Fig. 3:**
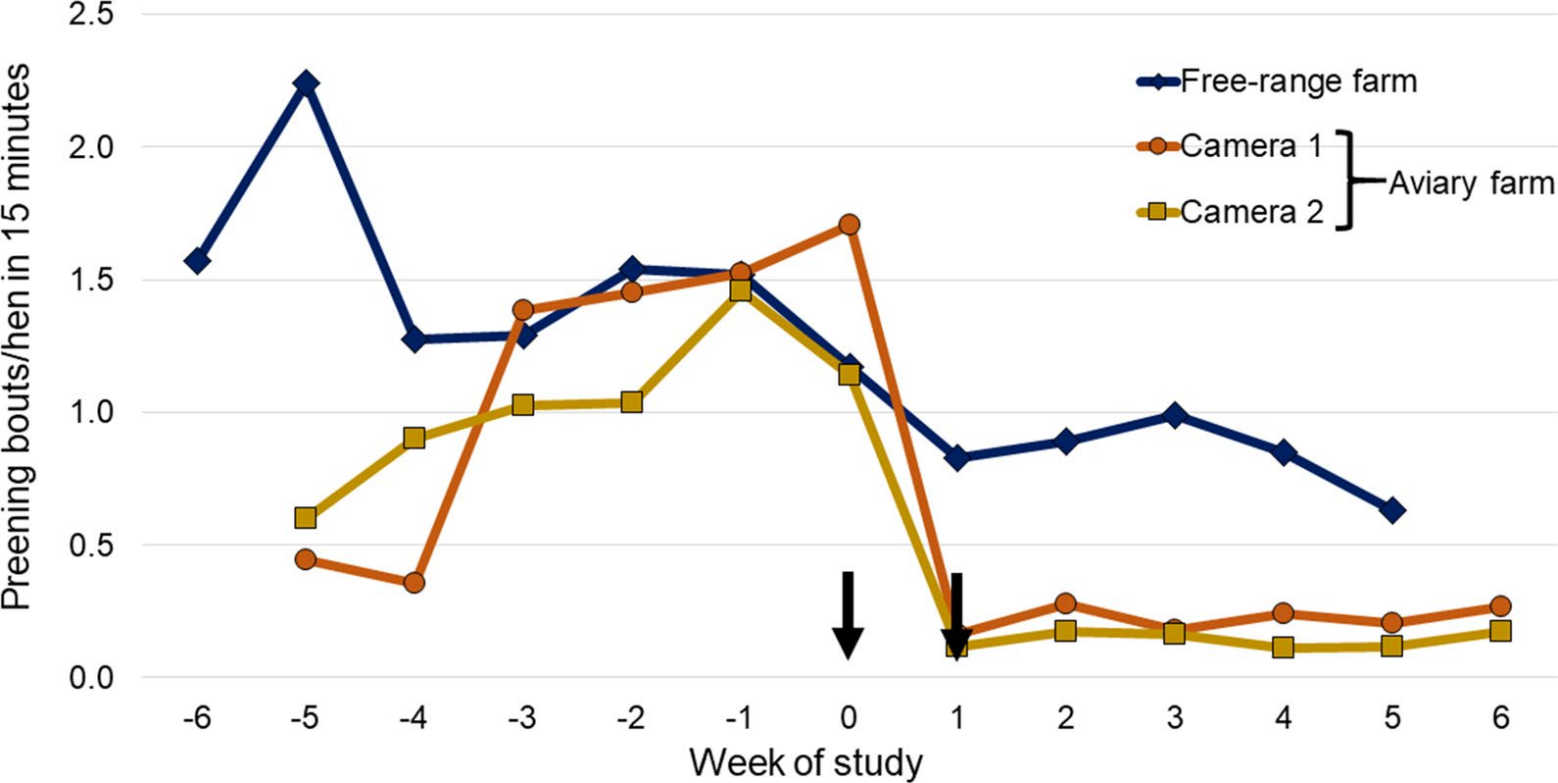
Nighttime hen preening activity (preening bouts per hen in a 15-min period) at each weekly assessment. Arrows indicate fluralaner administrations (Weeks 0 and 1)

**Fig. 4:**
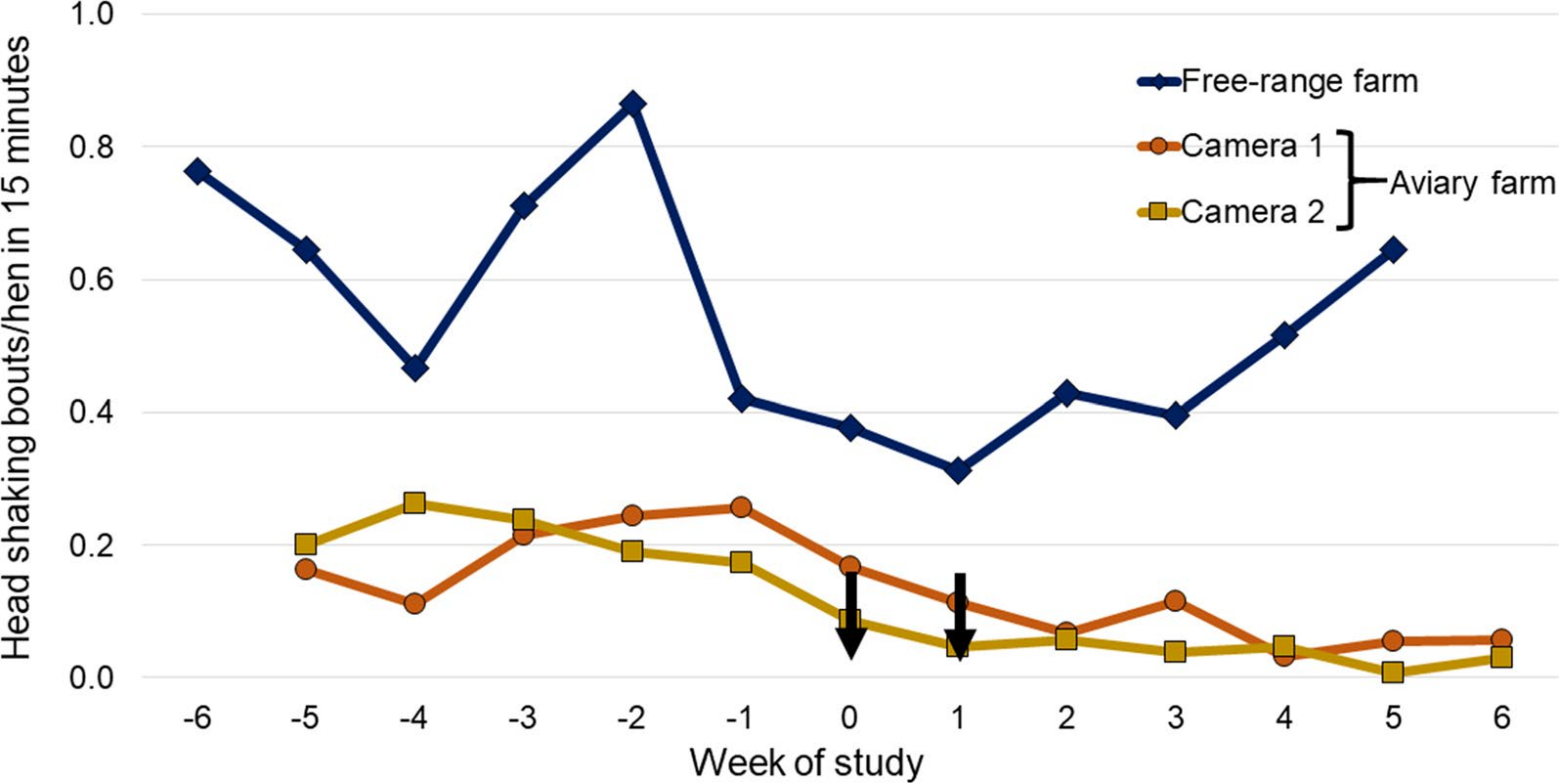
Nighttime hen head-shaking activity (bouts per hen in a 15-min period) at each weekly assessment. Arrows indicate fluralaner administrations (Weeks 0 and 1)

On both farms, fluralaner treatment and mite elimination were followed by significant reductions in nighttime activity (i.e., increase in the percentage of resting hens) (Fig. 2: aviary farm, *P* < 0.0001; free range farm, *P* = 0.0175). Following fluralaner treatment of birds on the aviary farm, the percentage of resting hens increased quickly, from 86.7% and 79.5% (Cameras 1 and 2, respectively) at Week − 2 to > 95% from Week 1 through the remainder of the study in both cameras. Significant post-treatment versus pre-treatment nighttime reductions were observed in both head shaking (aviary, *P* < 0.0001; free-range *P* = 0.0233) and preening (*P* = 0.0032; *P* = 0.0018) (Table 2; Figs. 3, 4). Nighttime body shaking (*P* = 0.0108), vertical wing shaking (*P* = 0.0002), head scratching (*P* = 0.0335) and gentle feather pecking (*P* < 0.0001) were also significantly reduced on the aviary farm, where there was no significant interaction of study phase and observation point (*P* = 0.6859). Daytime observations from the free-range farm found significant post-treatment versus pre-treatment reductions in head scratching (*P* < 0.0001), head shaking (*P* = 0.0492) and preening (*P* = 0.0012).

**Table 2:** Stress-associated behaviours of hens in which there was a significant improvement following fluralaner treatment

	Free-range farm		Aviary farm
	Night	Day	Night
Body shaking			*
Vertical wing shaking			***
Head scratching		***	*
Head shaking	*	*	***
Preening	**	**	**
Gentle feather pecking			***
Resting	*		***

Arrows indicate fluralaner administrations (Weeks 0 and 1)

Significant difference from pre-treatment value *P < 0.05; **P < 0.01; ***P < 0.001

### Physiological and haematological analyses

Corticosterone levels in egg yolk samples from the free-range farm were 9.3 (± 6.5) ng/ml prior to treatment (Week − 6), 17.7 (± 6.7) during the week of treatment (Week 0) and 17.6 (± 6.7) in samples collected in Week 6. The difference in corticosterone levels between Week 0 and Week 6 was not significant (*P* = 0.9321). On the aviary farm corticosterone in egg yolk and albumen and plasma increased at each assessment from pre-treatment through the week of treatment (Week − 1) to reach their highest levels at the final assessment (Week 5) (Table 3).

**Table 3:** Mean (± standard deviation) corticosterone concentrations in eggs collected from the free-range (Weeks − 6, 0 and 6) and aviary farms (Weeks − 6, − 1 and 6) and blood collected from hens on the aviary farm (weeks − 2, 0 and 6)

Week	Free-range farm	Aviary farm		
	Yolk (ng/g)	Yolk (ng/g)	Albumen (ng/g)	Plasma (pg/ml)
Pre-treatment	9.3 ± 6.5	5.9 ± 4.4	0.43 ± 0.06	16.2 ± 7.7
Treatment week	17.7 ± 6.7	7.2 ± 4.5	0.46 ± 0.04	23.2 ± 8.3
Post-treatment	17.6 ± 6.7	10.2 ± 5.5*^a^	0.5 ± 0.08**^a^	27.9 ± 10.3*^a^

Significant difference from pre-treatment value *P < 0.05; **P < 0.01

^a^Comparison *vs* Week − 1 value

Haematology results (only monitored on the aviary farm) revealed significant increases from Day 0 to Day 42 in erythrocytes, haematocrit, leukocytes, heterophils and lymphocytes (Table 4). The heterophil:lymphocyte ratio at Week 6 was significantly higher than at Week 0.

**Table 4:** Mean (± standard deviation) haematology results of blood samples collected from 40 randomly selected hens on the aviary farm

	Means			Comparison Day 0 *vs* Day 42
	Day − 14	Day 0	Day 42	
Erythrocytes (10^6^/μL)	2.3 (± 0.2)	2.2 (± 0.5)	2.4 (± 0.4)	*P* = 0.0256
Haemoglobin (g/dl)	7.9 (± 0.2)	7.8 (± 2.0)	8.1 (± 1.6)	*P* = 0.4474
Haematocrit (%)	17.6 (± 0.2)	15.3 (± 3.7)	17.5 (± 3.4)	*P* = 0.0111
MCV (fl)	77.2 (± 5.8)	71.0 (± 7.5)	72.5 (± 7.2)	*P* = 0.3978
MCH (pg)	35.2 (± 3.3)	34.7 (± 6.6)	33.3 (± 3.3)	*P* = 0.2646
MCHC (g/dl)	38.9 (± 1.8)	38.8 (± 2.4)	39.4 (± 2.5)	*P* = 0.2891
Leukocytes (10^3^/μl)	8.1 (± 1.1)	7.8 (± 1.1)	9.1 (± 1.5)	*P* < 0.0001
Heterophils (N)	3.7 (± 0.8)	3.7 (± 0.7)	5.7 (± 1.1)	*P* < 0.0001
Lymphocytes (N)	4.4 (± 0.7)	4.0 (± 0.7)	3.3 (± 3.9)	*P* < 0.0001
Ratio H/L	0.9 (± 0.2)	1.0 (± 0.2)	1.8 (± 0.5)	*P* < 0.0001

### Production assessments

The weekly average mortality rate did not significantly change with time on either the free-range farm (0.04% to 0.24%) (*P* = 0.3583) or the aviary farm (0.21% to 0.44%) (*P* = 0.6509). Consistent with the industry standard, the laying rate of the LSL hens on the free-range farm showed a significant decline as the birds aged (*P* = 0.0054), but this decline occurred at a lower rate (0.27% per week) than the published standard for LSL hens (0.42% per week) of the same age and time in production. Egg weights on this farm on Weeks − 6, 0 and 6 were 63.3, 65.9 and 66.6 g, an increase that was significant with time (*P* = 0.0008), and a mean increase of 0.28 g per week, while, according to the breed standard, an increase of 0.10 g per week would be expected. The rate of decline of the laying rate of the Lohmann Brown hens on the aviary farm (0.56% per week; *P* = 0.0288) was similar to the mean decrease of the standard laying rate expected for birds of the same age and breed. Although the egg weights of study hens on that farm (range 65.1 to 65.6 g) did not change significantly as the birds aged (*P* = 0.4065), throughout the study egg weights were approximately 3 g below the breed standard weight for hens of the same age.

## Discussion

This study provides further confirmation of laboratory and field studies showing that the rapid onset and high efficacy of fluralaner allows rapid control of the PRM in infested poultry flocks. On the free-range farm, the mean mite count at the end of the study was 1.4. Just a single mite was detected after Week 1 on the aviary farm, on which a calculation error resulted in administration of a dose rate 11% higher than recommended. As fluralaner efficacy on the free-range farm was equivalent to that on this farm, and as efficacy at the recommended dose rate has been consistently shown to approximate 100% on other layer farms, it can be concluded that this increase in dose rate did not affect the results, and there were no treatment-related adverse events [15–17]. Nonetheless, the error emphasizes the need for careful calculation of the appropriate product volume to ensure accurate delivery of the correct dose rate.

In the current study, the finding on both farms that mite elimination or near-elimination was followed by an improvement in nighttime hen resting behaviour, reductions in preening and head shaking and, on the aviary farm, head scratching aligns with similar findings from a recent study on an enriched cage farm in Spain [17]. Similarly, the current study and the Spanish study both align with earlier work showing that PRM infestation results in increases in those stress-related behaviours and provide further confirmation of the effects of PRM infestation in reducing bird welfare [5, 8]. As with the daytime findings in our study, the report from Spain also described daytime reductions in head scratching, head shaking and preening following mite elimination. Physiologically, stress-related behaviours have been linked to markers of somatic stress, including increases in plasma corticosterone with the potential to lower humoral immunity, and increases in the heterophil:lymphocyte ratio [17, 24]. However, in the current study blood and egg levels of corticosterone increased from pre-treatment to the week of treatment and increased further at the final assessments, as did the H:L ratio on the aviary farm. In the study of layer hens in Spain, post-treatment reductions in stress-related behaviours were accompanied by significant reductions in blood corticosterone levels and in the H:L ratio [17]. In that study, birds had been trained to accommodate handling, and the same birds were sampled on each occasion. In contrast, in the current study, birds received no such training and samples were collected from different birds on each occasion. The stress of restraint and blood collection from untrained birds may account for the increases in physiological stress markers seen in the current study. Perhaps linked to that finding is the observation that nighttime head shaking and preening on the free-range farm were generally greater than on the aviary farm, and the percentage of resting hens was generally lower. The increase in stress-related behaviours on the free-range farm could be a result of unrecognized stress factors affecting study birds. For instance, staff and management systems were different on the two farms, hens on the free-range farm may have been aware of predator threats, or stress may have arisen due to competition for access to feed or to differences between hen breeds on the two farms. Those potential non-PRM-related stressors may have reduced the observed treatment response, which was, nonetheless, still clearly present in the free-range birds, albeit not as marked as in the aviary birds. Thus, removing the irritant factor of an infestation may have reduced external stress behaviours, but other factors may have driven the stress-physiology findings.

Whether clinical or subclinical in appearance, anaemia is a well-established consequence of PRM infestation. A laying hen may lose 3% of its blood volume every night due to exposure to heavy infestations, while sub-acute anaemia up to death through severe anaemia has been reported [11, 25]. The significant increase in red blood cell counts and haematocrit from samples on the aviary farm indicate that removal of the PRM challenge by the fluralaner administrations resulted in a restoration of those blood values, providing further confirmation of the blood-draining impact of infestation.

The effects of PRM infestations on interfering with layer productivity have been documented in a number of studies. Affected parameters have included increases in hen mortality percentage and in the proportion of downgraded eggs and reductions in egg weight and laying rate [4, 9, 11, 16, 17]. There was no noticeable change in mortality rates between pre- and post-treatment periods on either farm. On the free-range farm, based on the industry standard for LSL hens, the expected decline in laying rate over time was lower than expected and the rate of increase in egg weights was greater than expected. This was less the case on the aviary farm where, throughout the observation period, the laying rate paralleled the industry standard for Lohmann Brown hens, while the rate of increase in egg weights remained below the breed standard, both before and after mite elimination. Overall, the results suggest that any production-impairing effects of the PRM infestation were removed by treatment. That conclusion is consistent with a report in which egg laying rates on seven of eight layer farms improved by up to 12.6% following PRM elimination by treatment with fluralaner compared with the laying rates observed in infested control birds that were either untreated or that received rescue treatments [16].

The primary objective of demonstrating the benefits of PRM elimination in reducing the frequency or duration of stress-related bird behaviours was achieved. Although production improvements were a secondary objective in this study, positive benefits were suggested by the results from the free-range farm, if not so clearly seen in results from the aviary farm. Importantly, the results of the current study provide further confirmation of the beneficial effects of PRM elimination on bird welfare.

## Conclusion

On two commercial poultry farms affected with heavy PRM infestations, two drinking water administrations of fluralaner, 7 days apart, largely eliminated mite challenge and reduced the incidence of stress-related layer hen behaviours. For the assessment of corticosterone a different surrogate than blood is recommended in untrained birds. Further work is needed to determine the productivity benefits suggested by the production data of treated birds.

## Data Availability

The datasets generated and analysed during the current study are available on reasonable request to MSD Animal Health Innovation, Schwabenheim Germany.

## Abbreviations

GCP: Good clinical practice
H:L: Heterophil:lymphocyte
PRM: Poultry red mite
VICH: Veterinary International Cooperation on Harmonisation
